# Do Frogs Get Their Kicks on Route 66? Continental U.S. Transect Reveals Spatial and Temporal Patterns of *Batrachochytrium dendrobatidis* Infection

**DOI:** 10.1371/journal.pone.0022211

**Published:** 2011-07-21

**Authors:** Michael J. Lannoo, Christopher Petersen, Robert E. Lovich, Priya Nanjappa, Christopher Phillips, Joseph C. Mitchell, Irene Macallister

**Affiliations:** 1 Indiana University School of Medicine, Terre Haute, Indiana, United States of America; 2 Naval Facilities Engineering Command Atlantic, Norfolk, Virginia, United States of America; 3 Naval Facilities Engineering Command Southwest, San Diego, California, United States of America; 4 Association of Fish and Wildlife Agencies, Washington, D. C., United States of America; 5 Illinois Natural History Survey, Institute for Natural Resource Sustainability, University of Illinois, Champaign, Illinois, United States of America; 6 Mitchell Ecological Research Service, Gainesville, Florida, United States of America; 7 United States Army Corps of Engineers, Construction Engineering Research Laboratory, Champaign, Illinois, United States of America; Institute of Marine Research, Norway

## Abstract

The chytrid fungus *Batrachochytrium dendrobatidis* (*Bd*) has been devastating amphibians globally. Two general scenarios have been proposed for the nature and spread of this pathogen: *Bd* is an *epidemic*, spreading as a wave and wiping out individuals, populations, and species in its path; and *Bd* is *endemic*, widespread throughout many geographic regions on every continent except Antarctica. To explore these hypotheses, we conducted a transcontinental transect of United States Department of Defense (DoD) installations along U.S. Highway 66 from California to central Illinois, and continuing eastward to the Atlantic Seaboard along U.S. Interstate 64 (in sum from Marine Corps Base Camp Pendleton in California to Naval Air Station Oceana in Virginia). We addressed the following questions: 1) Does *Bd* occur in amphibian populations on protected DoD environments? 2) Is there a temporal pattern to the presence of *Bd*? 3) Is there a spatial pattern to the presence of *Bd*? and 4) In these limited human-traffic areas, is *Bd* acting as an epidemic (i.e., with evidence of recent introduction and/or die-offs due to chytridiomycosis), or as an endemic (present without clinical signs of disease)? *Bd* was detected on 13 of the 15 bases sampled. Samples from 30 amphibian species were collected (10% of known United States' species); half (15) tested *Bd* positive. There was a strong temporal (seasonal) component; in total, 78.5% of all positive samples came in the first (spring/early-summer) sampling period. There was also a strong spatial component—the eleven temperate DoD installations had higher prevalences of *Bd* infection (20.8%) than the four arid (<60 mm annual precipitation) bases (8.5%). These data support the conclusion that *Bd* is now widespread, and promote the idea that *Bd* can today be considered *endemic* across much of North America, extending from coast-to-coast, with the exception of remote pockets of naïve populations.

## Introduction

One fifth of the world's amphibians may now be facing extinction [1], [2], [3]. In part these declines have been caused by the spread of the chytrid fungus, *Batrachochytrium dendrobatidis* (*Bd*) [4], which has been devastating amphibian populations on a global scale [2], [5], [6], [7], [8], [9], [10], [11]. In the United States, this pathogen can now be found from below sea level [12] to the highest elevations where amphibians occur [13], [14]. To date, however, most studies have been conducted locally, on single populations or within regions, and have often used different sampling protocols and analytical techniques [15], [16], [17], [18], [19], [20], [21]. The result is a piecemeal picture of what is most certainly a more widespread pattern [20], [22], [23], [24], [25], [26], [27], although there have been attempts to generalize across broader geographic areas [14], [28], [29], [30], [31], [32], [33], [34], [35].

Because of the variable nature of the available datasets (a situation we do not criticize), there have been questions about the occurrence and spread of *Bd*. In response, two general scenarios have been proposed that have strong empirical support [36], [37], [38]. In the first scenario, *Bd* is an *epidemic* (arising outside the population), spreading as a wave and wiping out individuals, populations, and species in its path. This has been well documented and is occurring in Central America, in eastern Australia, and in parts of California [35], [39], [40], [41], [42], [43], [44], [45]. The second scenario suggests that in certain regions of the world, such as North America, much of the spread of *Bd* occurred decades ago (when it was an epidemic) and that in these places it is now *endemic* (arising within the population) [35], [45], [46]. Indeed, *Bd* is now widespread throughout many geographic regions and is known to occur on every continent inhabited by amphibians (though some land masses and regions appear to remain naïve); therefore, this infection may be considered global [15], [16], [18], [38], [47], [48], [49], [50], [51], [52], [53], [54], [55], [56], [57], [58]. A third scenario, the *Bd* thermal optimum hypothesis, combines the first two hypotheses and has been more controversial. It suggests widespread benign *Bd* distribution has been triggered to lethality in regions by increased temperatures due to global warming [59], but this interpretation has been contentious [60].

Exploring the epidemic and endemic hypotheses requires, in part, broad-scale studies using standardized techniques. Further, due to the confounding factor of human disturbance on broad-scale patterns, it is best to examine low-impact (i.e. “natural”), or well-protected areas. Perhaps the most widely available habitats that remain “undisturbed” (a relative term, with perhaps the exception of the deep sea floor it is likely there are no longer any truly undisturbed environments left on earth [61]) in the United States today are United States Department of Defense (DoD) installations, which are secured as a matter of national interest. Military installations are protected against the indiscriminate human traffic experienced by parks, wildlife refuges, and other public areas. Moreover, following the tragic events of September 11^th^, 2001, access to these installations has been further limited, and in some cases severely restricted. DoD installations encompass over 12 million ha and occur throughout the United States, making continent-wide surveys possible. DoD lands are managed differently than typical surrounding landscapes, using ecosystem management techniques. Indeed, American military lands are thought to harbor the greatest concentrations of endangered and threatened species in the United States [62].

We conducted a transcontinental transect designed to assess the presence of *Bd* on military lands across the North American continent. DoD installations were sampled from west to east along U.S. Highway 66 (the “Mother Road”) from California into central Illinois, and continuing eastward from there to the Atlantic Seaboard along U.S. Interstate 64 (in sum from Marine Corps Base Camp Pendleton in California to Naval Air Station Oceana in Virginia, between 33° and 39° N latitude). We sampled across warm seasons, and used standardized collection and analytical techniques to address the following questions: 1) Does *Bd* occur in amphibian populations in these relatively undisturbed environments? 2) Is there a temporal (seasonal) pattern to the presence of *Bd*? 3) Is there a spatial pattern to the presence of *Bd*? and 4) In secured, limited-traffic areas of the country, is *Bd* acting as an epidemic (i.e., is there evidence of recent introduction and/or die-offs due to chytridiomycosis), or as an endemic (is it present without clinical signs of disease)?

## Materials and Methods

### Ethics Statement

This research was conducted under IACUC number 3-24-2008 issued by Indiana State University, and Scientific Purposes License Permit numbers 001641 (California Department of Fish and Game), SP783845 (Arizona Game and Fish), 3433 (New Mexico), 4621 (Oklahoma Department of Wildlife Conservation), 14113 (Missouri Department of Natural Resources), 09-0108 (Indiana Department of Natural Resources), SC0911102 (Kentucky Department of Fish and Wildlife), 031168 (Virginia Department of Game and Inland Fisheries). No animals were harmed while collecting *Bd* samples.

### Field Samples

**Figure 1:**
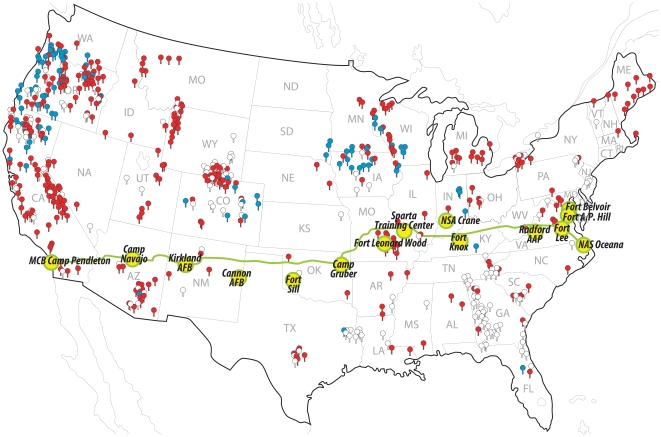
Department of Defense installations sampled in the present study. From California to Illinois, bases (yellow dots) were located near Route 66; from Illinois east to the coast, sites were chosen near Interstate 64 to hold latitude relatively constant (between 33° and 39° N). Our transect is shown overlain on a redrawn United States portion of the Global *Bd*-Mapping Project [14], with red pins indicating positive sites, white pins indicating negative sites, and blue pins indicating negative sites with sample sizes unknown.

In 2009, a total of 15 DoD installations were sampled as follows (Fig. 1; from west to east): Marine Corps Base Camp Pendleton in California, Camp Navajo in Arizona, Kirtland and Cannon Air Force Bases in New Mexico, Fort Sill and Camp Gruber in Oklahoma, Fort Leonard Wood in Missouri, Sparta Training Center in Illinois, Naval Support Activity Crane in Indiana, Fort Knox in Kentucky, and Radford Army Ammunitions Plant, Fort Lee, Fort A.P. Hill, Fort Belvoir, and Naval Air Station Oceana in Virginia. Each base was sampled three times: once in the spring/early summer (April, May, or the first week in June), once in mid-summer (July, August), and once in the late summer/fall (September, October), conforming to environmental conditions when *Bd* is most likely detectable [63]. Generally, three wetlands were sampled at each installation. Most sampling occurred at night, when amphibians are active, using dip nets. Captured amphibians were placed in new, individual plastic bags for processing and handling. Bags were discarded after one use; boots and nets were rinsed to clean off mud and debris, and sterilized with a dilute bleach solution between wetland sites.

Amphibians are the only known animate host for *Bd* [4], [45], [64], although zoospores can survive for up to 12 weeks under favorable (cool, moist) soil conditions [65], [66], [67], [68], [69]. Therefore our survey effort focused exclusively on amphibian populations. At each installation, postmetamorphic animals were sampled as they were encountered—because we were broadly interested in the presence of *Bd* we did not discriminate between salamanders and frogs. All animals were handled using sterile techniques and sampled using cotton, wooden-handled swabs. Swabs were rubbed while rolling the cotton over the body surface; five rubs each on the dorsum, flanks, ventrum, cranium, inguinal region, and the palmar/plantar surface of each foot for a total of 50 rubs [64], [70]. The head of the swab was then broken off in an individually labeled 0.5 ml free standing polypropylene screw cap microcentrifuge tube (Fisherbrand 02-681-333) and stored and shipped cold prior to analysis.

### Laboratory Analyses

Swabs were analyzed for *Bd* using conventional PCR (polymerase chain reaction) techniques [71], [72], [73]. Briefly, to extract *Bd* DNA from field samples, 1 ml of 70% ethanol was added to microcentrifuge tubes containing sample swabs and stored overnight at −20°C. Swabs were removed and the supernatant was centrifuged (16,000× g for 10 min). ATL-PK (Qiagen) tissue lysis buffer (200 ml) was added to the pelleted fraction and incubated overnight (55°C). To detect *Bd* zoospores, we used a nested PCR approach (18). Amplification products were visualized on a 3% agarose gel (Ameresco agarose 3:1 HRB). Presence or absence of a 300-bp band was compared against the EZ Load 100-bp molecular ruler (Bio-Rad) and a positive control. Negative controls were run with each sample. Samples were analyzed twice; if either sample tested *Bd* positive the animal was considered infected. Results were recorded on digital spreadsheets (Microsoft Excel).

### Temperature and Precipitation Data, and Statistics

Mean monthly and annual temperature and precipitation data for a 30-yr period (1971–2000) were obtained from stations near or at each base by searching National Oceanic and Atmospheric Administration (NOAA) databases (http://cdo.ncdc.noaa.gov/cgi-bin/climatenormals/climatenormals.pl).

We used Akaike's information criterion (AIC) [74], [75] to compare models (general linear mixed models; SPSS v. 17) fitting the pattern of *Bd* presence to four variables (season [S]—spring/early summer, mid-summer, and late summer/fall; geographic location [G]—east, central, west; mean seasonal rainfall [R]; and mean seasonal temperature) and each possible combination of each of the four variables (23 candidate models in all). Under this model selection framework, one model (see below) was clearly the best fit. However, because of the danger in underfitting models (leaving out models with important biological inferences) [75], we explored the next two models using a hypothesis testing framework. In particular, because only temperature data from among our datasets were normally distributed (Shapiro-Wilk normality test, Program R). We used nonparametric Kruskal Wallis tests (SPSS v. 17) to refine our examination of geographic location by comparing *Bd* infection prevalences, temperatures, and precipitation values between arid and temperate installations. Arid installations were located west of the 100^th^ meridian, where mean annual precipitation is <60 cm, and included Marine Corps Base Camp Pendleton, Camp Navajo, Kirtland Air Force Base, and Cannon Air Force Base. Temperate bases were Fort Sill, Camp Gruber, Fort Leonard Wood, Sparta Training Center, Naval Support Activity Crane, Fort Knox, Radford Army Ammunition Plant, Fort A.P. Hill, Fort Belvoir, Fort Lee, and Naval Air Station Oceana. Significance levels were set at p≤0.05.

## Results

### Installations

Each base was visited three times (Table 1). In April, Camp Navajo and Fort Sill were too cold for amphibian activity, and bases were visited but no amphibians were found and therefore no samples were collected. During the mid-summer and late-summer/fall trips the ponds at Fort Belvoir were dry and no amphibians were detected. At all other sites, during all other sampling times, animals were collected and sampled. In total, from all bases, during all visits, 1,306 amphibians were sampled for this project, 217 (16.6%) swabs tested positive for *Bd*. In general, the more arid the site the more difficult it was to detect amphibians, especially later in the year, and fewer animals were collected from these bases.

**Table 1:** Summary of percent *Bd*-positive amphibians detected at each DoD installation for each of the three 2009 sampling periods.

SamplingPeriod	Base														
	MCB CampPendleton	CampNavajo	KirtlandAFB	Cannon AFB	Fort Sill	Camp Gruber	Fort Leonard Wood	SpartaTrainingCenter	NSA Crane	Fort Knox	RadfordArmyPlant	FortA.P. Hill	Fort Belvoir	FortLee	NASOceana
**Spring/** **Early Summer**	5 15		0 4	4 21		5 27	35 60	40 60	28 60	16 60	3 19	14 47	7 18	3 28	11 36
	33%		0%	19%		18%	58%	67%	47%	27%	16%	30%	39%	11%	31%
**Mid-Summer**	0 2	0 34	1 36	0 31	0 12	0 32	2 60	10 60	0 60	5 60	8 24	2 20		1 24	0 22
	0%	0%	2%	0%	0%	0%	3%	17%	0%	8%	33%	10%		4%	0%
**Late Summer/** **Early Fall**	0 2	0 1	0 6	0 13	0 31	0 2	1 60	5 60	0 60	0 60	4 17	3 16		1 15	3 31
	0%	0%	0%	0%	0%	0%	2%	8%	0%	0%	24%	23%		7%	10%

Bases are arranged geographically, as they occur from west to east (Fig. 1).

We did not detect *Bd* at two bases, Camp Navajo, AZ and Fort Sill, OK. This was not due to sample size, per se. Thirty five samples were taken at Camp Navajo; 34 in July (mid-summer), 1 in September (late-summer/fall). At Fort Sill, a total of 43 samples were taken; 12 during June (mid-summer), 31 during September (late-summer/fall). This result could have been caused, in part, to a lack of samples during the spring/early summer sampling period (due to cold and snow), when the majority of positive samples at other bases were collected (see below).

**Figure 2:**
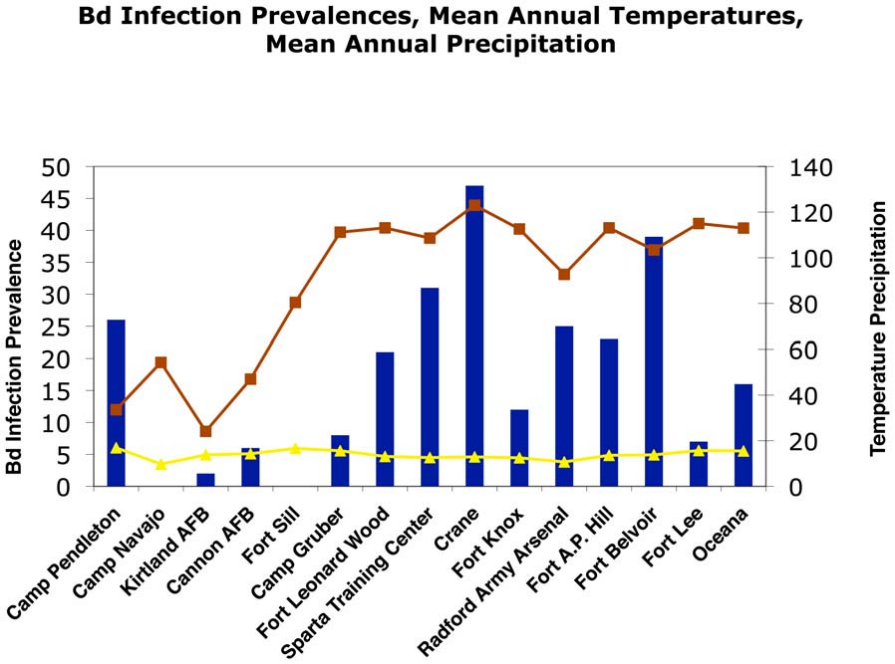
Prevalence (percentage) of *Bd*-positive samples by installation. Bases are arranged from west to east in the order they appear in Figure 1. Right side y-axis indicates both mean annual temperature (°C, yellow line) and mean annual precipitation (cm, red line). Note the generally low percentage of positive samples from the arid western installations.

*Bd* was detected at the remaining 13 bases (Table 1, Fig. 2). Infection prevalences among these sites ranged from 2% (1 of 46 samples positive) at Kirtland Air Force Base to 39% (7 of 18 samples positive) at Fort Belvoir. Other sites with high *Bd* prevalences included Sparta Training Center in Illinois (31%; 55 of 180 samples positive), Marine Corps Base Camp Pendleton in California (26%; 5 of 19 samples positive), and Radford Army Arsenal in Virginia (25%; 15 of 60 samples positive). Sparta Training Center had the highest absolute number of positive samples (55), Fort Leonard Wood had the second highest (38).

### Species

Fifteen of the 30 amphibians species sampled tested positive for *Bd*. Species infected covered a wide phylogenetic range (Table 2) including: four species of plethodontid salamanders (*Desmognathus fuscus*, *Eurycea cirrigera*, *Eurycea longicauda*, and *Pseudotriton ruber*), three species of toads (*Anaxyrus americanus, Anaxyrus fowleri, Anaxyrus woodhousii*), five hylid species (*Acris blanchardi, Acris crepitans*, *Hyla cadaverina*, *Hyla chrysoscelis*, and *Pseudacris crucifer*), and three ranid species (*Lithobates catesbeianus, Lithobates clamitans,* and *Lithobates sphenocephalus*). At no point during this study did we observe moribund amphibians.

**Table 2:** A list of species sampled for the presence of Bd, organized by families (bold); salamanders followed by frogs.

Ambystomatidae
*Ambystoma maculatum*
*Ambystoma mavortium*
*Ambystoma tigrinum*
Plethodontidae
Desmognathus fuscus*
Eurycea cirrigera*
Eurycea longicauda*
Pseudotriton ruber*
Salamandridae
*Notophthalmus viridescens*
Bufonidae
Anaxyrus americanus*
Anaxyrus fowleri*
*Anaxyrus punctatus*
*Anaxyrus terrestris*
Anaxyrus woodhousii*
Hylidae
Acris blanchardi*
Acris crepitans*
Hyla cadaverina*
Hyla chrysoscelis*
*Hyla cinerea*
*Hyla femoralis*
*Hyla squirella*
*Hyla versicolor*
Pseudacris crucifer*
*Pseudacris regilla*
*Pseudacris triseriata*
Microhylidae
*Gastrophryne carolinensis*
Ranidae
*Lithobates blairi*
Lithobates catesbeianus*
Lithobates clamitans*
*Lithobates palustris*
*Lithobates sphenocephalus*

Asterisks indicate species where at least one specimen tested positive. Thirty species were tested, which represents about 10% of the species found in North America. Frog species are disproportionately representented.

### Temporal Patterns (Seasonality)

**Figure 3:**
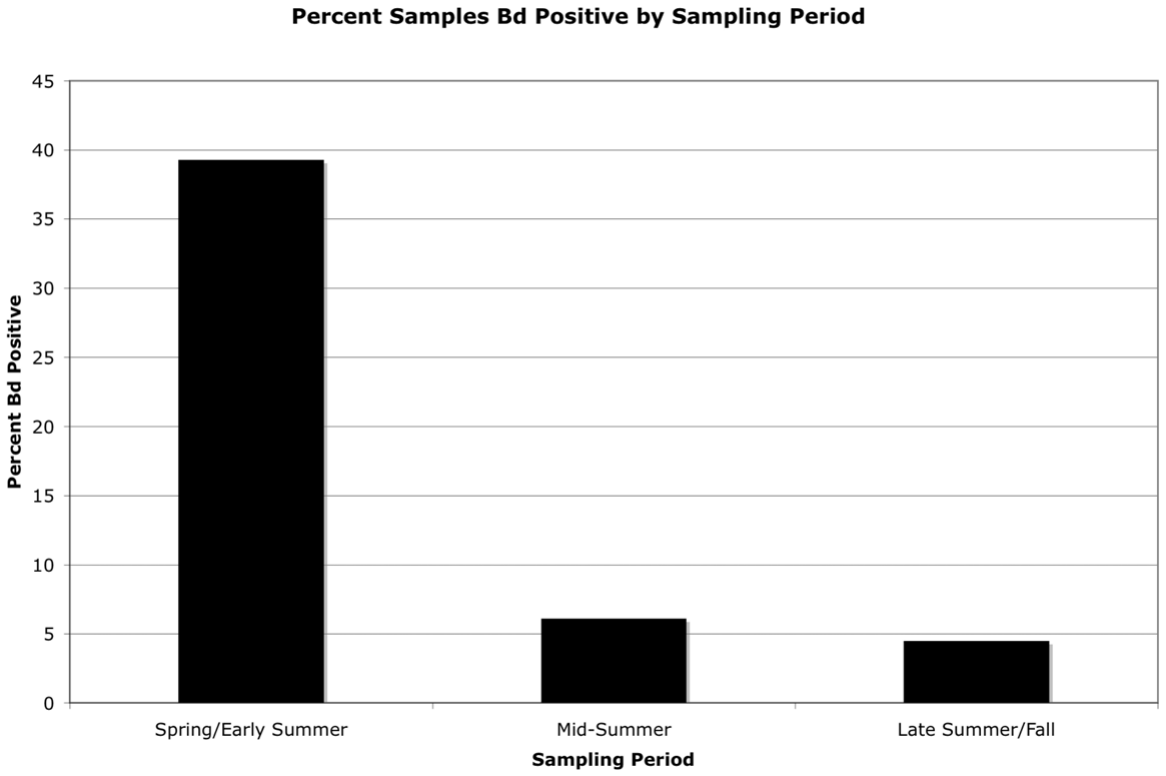
Temporal pattern (seasonality) of *Bd* infection incidence across all installations. Note the strong tendency for the highest incidences to occur during the spring/early summer sampling period, followed by a precipitous drop off during the mid- to late-summer and fall.

Among 23 candidate models based on season, geographic location, mean seasonal rainfall, and mean seasonal air temperature examined individually and in combination, seasonality produce the lowest AICc score (Table 3). We agree there was a strong seasonal component to our results (Table 3, Fig. 3). During the spring/early-summer sampling period, 39.3% of all samples were positive. This number dropped to 6.1% for mid-summer samples, and to 4.5% for late-summer/fall samples. Most bases followed this pattern (Table 1) including Marine Corps Base Camp Pendleton, Cannon Air Force Base, Camp Gruber, Fort Leonard Wood, Sparta Training Center, Naval Support Activity Crane, Fort Knox, and Fort Lee. Camp Navajo and Fort Sill had no positive samples and Fort Belvoir had animals collected only in the spring/early summer period, and therefore these bases had no temporal pattern of infection. Radford Army Arsenal had 15.8% positive in spring/early summer, 33.3% positive samples in mid-summer, and 23.5% positive in late-summer/fall. Three East-Coast installations—Fort A.P. Hill, Fort Lee, and Naval Air Station Oceana—had higher percentages of animals infected during the late-summer/fall than in the mid-summer period.

**Table 3:** AIC_C_ scores for the top six (out of 23) candidate models.

Model	k	AICc	ΔAICc	ωi	Evidence Ratio
S	3	-47.63	0.00	0.77	1.00
S+R	4	-42.51	5.11	0.06	12.89
T	2	-42.51	5.12	0.06	12.90
S+T	4	-41.19	6.43	0.03	24.95
T+R	3	-40.82	6.80	0.03	29.99
S+T+R	5	-39.85	7.77	0.02	48.69

Terms included in the models are the effects of season (S; spring/early summer, mid-summer, late summer/fall), geographic location (G; five eastern bases, five central bases, five western bases), mean annual rainfall (R) and mean annual air temperature (T). The seasonal model was clearly the best (lowest AICc value), followed by season/rainfall and temperature models. Headings: k  =  number of parameters; AICc  =  modified AIC criterion; ΔAICc  =  rescaled AIC; ωI  =  Akaike weight; Evidence Ratio  =  model comparison.

### Spatial Patterns

Among candidate models, seasonality plus mean annual rainfall produced the second lowest AICc score (Table 3). While this model was much less predictive, aridity did appear to have a negative effect on *Bd* prevalence. Five of the six sites with the lowest prevalences (Camp Navajo, 0%; Fort Sill, 0%; Kirtland Air Force Base, 2%; Cannon Air Force Base, 6%, and Camp Gruber, 8%) occur in the arid southwest (Arizona, New Mexico) or on or near the Great Plains (Oklahoma); the exception was Fort Lee (7%) in Virginia. Remaining sites occur in coastal areas, or inland areas that receive higher levels of precipitation (Table 4). A second way we explored this trend was to compare the data for the western arid bases (Marine Corps Base Camp Pendleton, Camp Navajo, Kirtland Air Force Base, and Cannon Air Force Base) with data for the eastern temperate sites. The prevalence of positive samples for the arid installations was 8.5±11.7% (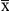
±95% C.I.)); the prevalence of positive samples for the eastern temperate sites was 20.8±8.4%. This difference was statistically significant (Kruskal-Wallis, p = 0.027). Precipitation levels were also different between the western arid and eastern temperate bases (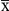 = 39.5±13.2 vs. 107.8±11.8 cm annually; Kruskal-Wallis, p = 0.002), although temperatures were not (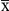 = 13.7±2.9 vs. 13.9±1.1°C; Kruskal-Wallis, p = 0.3).

**Table 4:** Mean annual precipitation (cm) and temperature (°C) at each of the fifteen DoD installations sampled.

Installation Name	Mean Annual Precipitation	Mean Annual Temperature
MCB Camp Pendleton	33.6	16.9
Camp Navajo	54.3	9.7
Kirtland AFB	24.1	13.8
Cannon AFB	47	14.3
Fort Sill	80.4	16.6
Camp Gruber	111.2	15.6
Fort Leonard Wood	113.1	13.1
Sparta Training Center	108.6	12.6
NSA Crane	123	12.9
Fort Knox	112.6	12.5
Radford	92.7	10.7
Fort A.P. Hill	113.1	13.6
Fort Belvoir	103.4	13.8
Fort Lee	115	15.7
NAS Oceana	113	15.5

These data are plotted against *Bd* infection rates at each base in Figure 2.

## Discussion

We used standardized collection and analytical techniques to address the following questions: 1) Does *Bd* occur in amphibian populations in these relatively undisturbed environments? 2) Is there a spatial pattern to the presence of *Bd*? 3) Is there a temporal pattern to the presence of *Bd*? and 4) In secured, limited-traffic areas of the country, is *Bd* acting as an epidemic (i.e., is there evidence of recent introduction and/or die-offs due to chytridiomycosis), or as an endemic (is it present without clinical signs of disease)?

Before we formally consider these questions, we offer some background on the life history and physiological ecology of *Bd*. The life history of *Bd* is composed of two stages: a thallus (body), which is present in amphibian skin and free-living zoospore, which is flagellated and motile in aquatic environments [76]. Zoospores can swim about 2 cm [77] and infect keratinizing squamous epithelial cells [78]. Favorable environments, where the infection can spread, are cool and wet. Hot and dry environments are considered hostile, and temperatures >25°C may assist infected amphibians in clearing the infection [76], [79], [80]. Given this, across amphibian species, behavioral and life history features of the animal, and ecological features of the geographic region (ecoregion), will affect the course and extent of *Bd* infection [81], [82], [83], [84].

In the present study, *Bd* was detected in 13 of 15 DoD installations, spanning the width of the North American continent. Fifteen of 30 amphibian species sampled tested positive for *Bd*. There were both spatial and temporal patterns to *Bd* prevalence, as follows.

### *Bd* is found in the highly secure environments of U.S. DoD installations

In aggregate, the data for all bases over all three sampling periods (spring/early-summer, mid-summer, late-summer/fall) show a 16.6% prevalence of *Bd* infection. *Bd* was found in all but two installations, Camp Navajo in Arizona and Fort Sill in western Oklahoma. Lack of *Bd* detection on these bases may be the result of insufficient sampling during the first sampling period due to inclement weather (cold/snow). Amphibians were not active during the first sampling period at either of these bases and spring and early-summer was the time when *Bd* was most likely to be detected (78.5% of our positive samples came from this first sampling period; see below).

During this study we sampled about 10% (30) of all known United States amphibian species and found *Bd* in half of them, including four plethodontids, three bufonids, five hylids, and three ranids (Table 2). While *Bd* absence in the remaining species may be due to inherent resistance [85], [86] or ecological avoidance [81], it is most probable that in cases of no detection, individuals sampled happened to be negative, or to test negative at the time of sampling. It is likely that all amphibian species are susceptible to *Bd* infection, although species-specific variation in susceptibility has been shown [87], as has intraspecific variation in susceptibility [25]. Several of the species that tested *Bd* positive have tested positive in other studies; salamanders and ranids, including American Bullfrogs (*Lithobates catesbeianus*), may be carriers of this infection [88], [89], [90], [91].

### There is a temporal (seasonal) pattern to the presence of *Bd*

There was a strong temporal component to our dataset (Table 1; Fig. 3). In total, 78.5% of all positive samples came in the first (spring/early-summer) sampling period, and broken out by sampling period, the percent positive samples were 39.3% (168 of 427), 6.1% (29 of 477), and 4.5% (17 of 374). The data for the majority of bases (Marine Corps Base Camp Pendleton, Cannon Air Force Base, Camp Gruber, Fort Leonard Wood, Sparta Training Center, Naval Support Activity Crane, Fort Knox, and Fort Lee) followed a temporal pattern, but the data from some bases did not (Table 1). For example, three bases had no pattern of infection: Camp Navajo and Fort Sill had no *Bd* positive samples and Fort Belvoir had positive samples only in the spring/early summer period. Other bases had infection patterns that differed seasonally: Radford Army Arsenal had 15.8% positive in spring/early summer, 33.3% positive samples in mid-summer, and 23.5% positive in late-summer/fall. Interestingly, and perhaps reflecting more favorable conditions for *Bd* (high moisture), three East-Coast installations—Fort A.P. Hill, Fort Lee, and Naval Air Station Oceana—had higher percentages of animals infected during the late-summer/fall than in the mid-summer period (Table 1). Overall, our data suggest a strong seasonal component to *Bd* infection, with the earliest sampling period showing the greatest incidence (Fig. 3).

Seasonality in *Bd* incidence has been previously demonstrated [27], [92], [93], [94]. As summer proceeds, *Bd*-positive frogs appear to lose their infection [66], [77], [79], [84], [95]. It is also true that infected animals can develop chytridiomycosis and die, and thus be lost to later surveys. Just as we suggest the spatial pattern of *Bd* presence is due to variations in moisture levels (with moisture promoting infection incidence) we suggest the temporal (seasonal) pattern is due to moisture availability, with *Bd* present at the highest incidences during the wettest times of the year. Corroborating this hypothesis, our data suggest that *Bd* prevalence is lower in arid areas (xeric deserts and the arid Great Plains) and during drier times of the year (mid- to late-summer and fall). Temperature may be a covariate, with cooler temperatures promoting the infection, although in our study, by minimizing variation in latitude among sites in the transect [96], we also minimized, as much as possible in a continental transect, temperature differences.

### There is a spatial pattern to the presence of *Bd*

The eleven eastern temperate DoD installations had significantly higher prevalences of *Bd* infection (20.8%) than the four bases situated in the arid western ecosystems (8.5%). *Bd* went undetected at one of these bases (Camp Navajo); two arid bases each had single-digit levels of detection (Kirtland Air Force Base, 2%; Cannon Air Force Base, 6%). Marine Corps Base Camp Pendleton was the one exception. It had a 26% prevalence, but this installation is characterized by maritime Mediterranean climate, with more moisture than inland installations. Fort Sill was the second exception; *Bd* was not found on this eastern Oklahoma base. *Bd* is known to favor cool, moist conditions [38], [97]. It therefore follows that warm and dry (i.e., arid) conditions may inhibit this pathogen. Our data are consistent with this interpretation (Fig. 2). Further, animals that bask have been shown to have an increased resistance to chytridiomycosis [79], [80]. We find no evidence that particular species are driving geographic trends.

Our data both support and augment data on known distributions of *Bd* in the United States [14] (Fig. 1). Our data support the observations that *Bd* is widespread in California and common across portions of the southwest and throughout the eastern half of the country. The relatively few positive samples previously reported from the Great Plains may in part be due to the relatively few samples recorded from this region [14] (Fig. 1). In our study, positive samples from New Mexico, Oklahoma, and Illinois expand upon the sparse reporting from these states. Positive samples from Ft. Knox confirm the presence of *Bd* in Kentucky.

### Our results suggest *Bd* may be endemic across much of the United States middle latitudes

These data lend support to the conclusion [46] that *Bd* is now widespread across much of North America. Samples from DoD installations located in mesic habitats show a range of *Bd* infection from 7% (5/67) positive samples at Fort Lee, in Virginia, to 39% positive samples at Fort Belvoir. This spatial pattern—from coast-to-coast—supports the assertion [46] that *Bd*, while once likely *epidemic*, today is *endemic* across much of the United States. Further, the phylogenetic range of species that tested positive—plethodontid salamanders in three genera, bufonid toads, treefrogs in three genera, and ranid frogs—suggests that this infection has been associated with aquatic ecosystems long enough to infect numerous taxonomic groups.

The exceptions to this generalization of endemism are two arid bases, Camp Navajo in Arizona and Fort Sill in Oklahoma. These installations warrant further study; there would be greater confidence with the conclusion that *Bd* is absent at these bases had we sampled large numbers of animals during the spring/early-summer period. Our data do not distinguish between “*Bd* present but not detected” and “*Bd* absent.” There are known to be pockets of wilderness, for example in regions of the Sierra Nevada that *Bd* has yet to reach [45]. At these places, when *Bd* arrives it is predicted to be an *epidemic* infection, and we suspect amphibian extirpations will follow. Camp Navajo and Fort Sill may be examples of such *Bd*-negative outposts, but further study is needed.

Our results contribute to the recent and widespread literature being generated on the spatial patterns of *Bd* distribution tied to assessing risks and identifying refugia [98], [99], [100], [101], [102]. In particular, these authors suggest that amphibian species with populations inhabiting the drier interior of the United States may be less at risk than populations inhabiting more mesic regions. Attempts to ameliorate the effects of *Bd* in the field are being proposed [103]. Our data also suggest that broad geographic surveys, whether they be current or historical, be interpreted in light of seasonality in collection times. For example a directional transect beginning in one region and ending sometime later at a distant region, may interpret a geographic trend, when in fact the cause might be a seasonal trend.
